# Dental Care for Patients with End-Stage Renal Disease and Undergoing Hemodialysis

**DOI:** 10.1155/2018/9610892

**Published:** 2018-11-13

**Authors:** Fulvia Costantinides, Gaetano Castronovo, Erica Vettori, Costanza Frattini, Mary Louise Artero, Lorenzo Bevilacqua, Federico Berton, Vanessa Nicolin, Roberto Di Lenarda

**Affiliations:** ^1^Unit of Periodontology and Dental Hygiene, School of Dental Sciences, Department of Medical, Surgical and Health Sciences, University of Trieste, Italy; ^2^Division of Nephrology and Dialysis, “Maggiore” University Hospital, Trieste, Italy

## Abstract

Chronic renal failure is a progressive disease characterized by a gradual destruction of nephrons and a consequent reduction of kidney function. End-stage renal disease (ESRD) necessitates renal replacement therapy as peritoneal dialysis, hemodialysis, or transplantation. Patients affected by ESRD or in hemodialysis are at risk for developing a number of comorbidities including hypertension, anemia, risk of bleeding, susceptibility to infection, medication side effects, and oral manifestations associated with the disease itself and with hemodialysis treatment. In this context, oral diseases represent a potential and preventable cause of poor health outcomes in people with ESRD due to their relation to infection, inflammation, and malnutrition. The aim of this article was to review ESRD and hemodialysis-associated manifestations and to describe the dental operative protocols for patients awaiting kidney transplantation in light of the most recent literature.

## 1. Introduction

Oral health represents a potential determinant of health outcomes in patients with end-stage renal diseases (ESRD). Adults with ESRD have more severe oral diseases than the general population, and dental conditions such as caries, periodontitis, and poor oral hygiene are associated with increased mortality. Oral pathologies are associated with inflammation and malnutrition, which may accelerate cardiovascular events in ESRD [1]. Furthermore, in the context of kidney transplantation, infection from a dental source is a potential threat for both organ transplant candidates and recipients since dental disease is a ubiquitous condition and is also likely to be more severe and untreated in the transplant population [2].

The present work reviews the literature on general and specific considerations regarding the dental treatment of ESRD patients in the pretransplant phase, considering the systemic problems and treatment that may interfere with clinical practice.

## 2. Chronic Renal Disease: General Considerations

Chronic renal failure is a progressive disease characterized by a gradual destruction of nephrons and a consequent reduction of kidney function occurring over a few months or years [3]. As this process develops, the glomerular filtration rate (GFR) falls while serum levels of urea rise. GFR is the rate at which an ultrafiltrate of plasma is produced by glomeruli per unit of time and is the best estimate of the number of functioning nephrons or functional renal mass [4]. Normal GFR values are approximately 120–130 mL/min/1.73 m^2^ and vary according to age, gender, and body size [5]. All individuals with a GFR <60 mL/min/1.73 m^2^ for 3 months are classified as having chronic kidney disease, irrespective of the presence or absence of kidney damage. The reduction of GFR is usually measured by creatinine clearance (CC), which gives an acceptable approximation of the value of GFR^5^. In clinical practice, the CC can be indirectly assessed through serum creatinine (normal values 0.5–1.4 mg/dl) using several formulas such as Chronic Kidney Diseases Epidemiologic Collaboration (CKD-EPI) equation or the Modification of Diet in Renal Disease (MDRD) equation.

The progressive loss of renal function can be staged through clinical, instrumental, and laboratory findings in the following.

Stage 1: slightly diminished function—kidney damage with normal or relatively high GFR (≥90 mL/min/1.73 m^2^). Kidney damage is defined as pathological abnormalities or markers of damage, including abnormalities in blood or urine tests, biopsy, or imaging studies. Stage 2: mild reduction in GFR (60–89 mL/min/1.73 m^2^) with kidney damage. Stage 3: moderate reduction in GFR (30–59 mL/min/1.73 m^2^). Stage 4: severe reduction in GFR (15–29 mL/min/1.73 m^2^)—preparation for renal replacement therapy. Stage 5: established kidney failure or end-stage renal disease (ESRD) (GFR <15 mL/min/1.73 m^2^) requiring permanent renal replacement therapy (RRT).

In 2004, KDIGO (Kidney Disease: Improving Global Outcomes) added a specification regarding the concomitant presence of a replacement therapy using the letter “T” for transplanted patient and “D” for dialysis [6].

The two main causes of ESRD are hypertension and diabetes mellitus, both of which have a deleterious impact on the cardiovascular system and renal apparatus of the patients before and after transplantation. Particularly, cardiovascular diseases (atherosclerosis) represent the main cause of death in renal transplant recipients. Other causes of ESRD are glomerulonephritis, chronic pyelonephritic urologic disorders, and autoimmune diseases [7]. The most common cause of death in ESRD patients is cardiac arrest, followed by infection and malignancy [8].

Treatment of chronic renal insufficiency includes dietary modifications and correction of systemic complications [8]. ESRD, also known as the uremic syndrome, necessitates renal replacement therapy as dialysis or transplantation [4].

While stages 1 to 3 do not pose any contraindications for routine dental treatment, patients with advanced kidney disease (stages 4-5) require special considerations, most importantly regarding hypertension, anemia, risk of bleeding, infection and medication used, and oral manifestations associated with the disease itself and with hemodialysis treatment [9].

## 3. ESRD and Dialysis

ESRD is characterized by diminished endocrine and metabolic functions of the kidney with subsequent retention and accumulation of toxic metabolites. The blood pressure is increased due to fluid overload and production of vasoactive hormones via the renin-angiotensin system, increasing the risk of developing congestive heart failure. As chronic kidney disease progresses, the ability to produce renal hydroxylated 1,25 vitamin D diminishes and 1,25 vitamin D deficiency ensues [10]. Erythropoietin synthesis is also decreased, potentially leading to anemia. Furthermore, anemia increases the hemorrhagic tendency in uremia due to qualitative platelet dysfunction. Platelet dysfunction is due to both decreased platelet aggregation and impaired platelet adhesiveness and is one of the main determinants of uremic bleeding. This impairment is multifactorial and includes defects intrinsic to the platelet as well as abnormal platelet-endothelial interaction. Uremic toxins and anemia also play a role. Correction of platelet dysfunction is desirable in patients who are actively bleeding or who are about to undergo a surgical procedure (e.g., renal biopsy). Platelet dysfunction in the uremic patient can be controlled with the administration of desmopressin, an analog of antidiuretic hormone with little vasopressor activity, by the administration of conjugated estrogens, or infusion of cryoprecipitate that can shorten the bleeding time in many uremic patients [11–13].

The ESRD patient also presents an immunodeficient state caused by altered cellular immunity associated with malnutrition, susceptibility to bacterial infection, and a diminished ability to produce antibodies [14].

A final consideration with respect to pharmacotherapy is that most drugs are at least partially excreted via the kidney. With renal dysfunction, the distribution, metabolism, bioavailability, and rate of excretion of the drug are altered, and an adjustment of the dosage by amount or by frequency is required [5].

With advanced renal disease, aggressive measures such as dialysis must be taken. Dialysis is a life-saving intervention that has significantly prolonged life expectancy in young patients. Hemodialysis is allowed by a machine (dialyzer) that contains semipermeable membranes. These membranes permit the passage of excessive fluid and wastes. Arteriovenous shunts or fistulas achieve the access to the bloodstream. The continuous accumulation of toxic products in ESRD requires that the patient undergoing hemodialysis receive the treatment for approximately three-four hours a day, three times a week. It is important to point out out that the efficiency of this process is much lower than a functioning kidney, hence ESRD patients on hemodialysis are in a constant state of kidney failure and hyperuremia. The constant presence of the uremic syndrome even in hemodialyzed patients has been forwarded as a primary contributor to the many systemic complications seen in these patients.

Peritoneal dialysis (PD) offers greater independence; access to the peritoneum is achieved by a catheter through the abdominal wall into the peritoneum that serves as a membrane able to filter the catabolic products from the local vessels [9]. Although hemodialysis and PD correct many of the hematologic dysfunctions associated with uremia, several issues already present in ESRD are exacerbated during the treatment. Hemodialysis requires the use of anticoagulant in the form of regional or systemic heparin to maintain access patency and facilitate the filtration of toxic blood compound such as urea through the dialysis membrane [4]. Heparinization associated with mechanical trauma to platelets can reduce the total platelet number and increase hemorrhagic risk. This tendency is worsened by the already present capillary fragility and anemia. Furthermore, patients tend to have hypertension attributed to salt and water retention and to activation of the rennin-angiotensin-aldosterone [15, 16].

Sudden death is the most common cause of death in dialyzed patients but also infection is a frequent cause of morbidity and mortality in patients receiving hemodialysis therapy. Due to the vascular access, patients are at increased risk of endarteritis and endocarditis that occurs in about 2.7% of patients during hemodialysis and in 9% of those who have an infection of the vascular access [17].

## 4. Oral Manifestations

It has been estimated that 90% of chronic renal failure patients present oral symptoms. However, with the improvements of hemodialysis technology many of the oral manifestations of renal failure and uremia described below are less commonly seen [4].

Oral manifestations involve mucosal and glandular tissues, the gingival and the periodontal apparatus, the maxillary and mandibular bone, and finally the dental status.

Primarily, in regard with the mucosal and glandular involvement, the most common oral finding in dialyzed patient is pallor of the mucosa due mainly to anemia (reduced erythropoietin synthesis) [5, 15]. Bleeding tendency in these patients is sustained by alterations in platelet aggregation and renal anemia [15, 18]. Also, hemodialysis predisposes to ecchymoses, petechiae, and hemorrhage in the oral mucosa [5, 19, 20].

Xerostomia denotes the subjective sensation of dry mouth and is related to the overall volume status of the patients who are discouraged from drinking excess fluids and who are often prone to retrograde parotitis [9, 15, 20, 21]. In association with xerostomia, one third of hemodialyzed patients present a characteristic halitosis called “uremic fetor” and a metallic taste due to high urea content in saliva and its breakdown in ammonia [5, 9]. Also, the patient can perceive altered sweet and acid flavors due to high levels of urea in saliva and to the presence of dimethyl and trimethyl amines. A burning sensation of lips and tongue and an enlarged tongue sensation may be additional symptoms noted by dialyzed patients [5]. An important problem is represented by uremic stomatitis, which is a relatively uncommon oral complication of unknown etiology [5, 18]. The lesions consist of localized or generalized erythematous areas covered by pseudomembranous exudates that can be removed, leaving an intact or ulcerated mucosa. The lesions are commonly painful and most often appear on the ventral tongue and anterior mucosal surfaces. They usually heal spontaneously with resolution of the underlying uremia and after lowering of the blood urea nitrogen (BUN) level [9]. However, in order to promote the healing of the lesions, gargling with 10% hydrogen peroxide 4 times a day can be recommended [5, 19]. Angular cheilitis has been reported in more than 4% of patients receiving hemodialysis, and lichenoid disease may arise associated with antihypertensive medication [5, 8]. Secondarily, pointing the attention on the periodontal health, patients undergoing hemodialysis generally have a poor objective periodontal status verified by mean CPITN (Community Periodontal Index of Treatment Needs), and deposit of calculus and plaque may be increased [5, 8, 22, 23]. Studies have shown that dental care in patients undergoing hemodialysis is neglected, and that they brush and floss infrequently [15]. Results of a study by Naugle et al. suggested that 100% (*n* = 45) of the individuals undergoing renal dialysis presented with some form of periodontal disease [24]. Moreover, diabetic nephropathic patients show deeper periodontal pockets compared with ESRD nondiabetic patients [25]. Also, the need for surgical treatment of periodontitis is significantly higher in patients awaiting kidney transplant compared with patients not undergoing organ transplantation [26]. Periodontitis itself contributes to systemic inflammation and has been associated with adverse hemodialysis outcomes including mortality [27]. The accelerated periodontal disease with pocket formation, gingival recession, and bone and tooth loss is due not only to inadequate oral hygiene and inflammatory disease burden but also to renal osteodystrophy [5, 15].

Maxillary and mandibular bone tissue is interested by renal osteodystrophy that results from disorders in calcium, phosphorus, and vitamin D metabolism and from increased parathyroid activity. Oral manifestations of renal osteodystrophy include tooth mobility, malocclusion, pulp stones, enamel hypoplasia, bone demineralization, decreased trabeculation of cancellous bone, decreased thickness of cortical bone, radiolucent giant cell lesions, jaw fracture (spontaneous or after dental procedures), and abnormal bone healing after extraction [5, 9, 20]. To avoid hypovitaminosis D and its consequences, it is therefore necessary to administer calcitriol or its analogs to compensate for the compromised production of 1,25 vitamin D, which occurs in the later stages of chronic kidney disease (beyond stage 3) so that the classical functions of hormonal 1,25 vitamin D may be addressed [10].

Finally, in regard with dental tissue involvement, a lower rate of caries has been observed. This finding can be explained by the possible antibacterial effect of a higher urea concentration in saliva that inhibits plaque and bacteria development [9, 15]. The antibacterial effect has been attributed to the increase of pH due to urea hydrolization by saliva, which suggests a protective function against caries [5, 16]. Dental erosions due to frequent regurgitation resulting from the nausea associated with hemodialysis treatments and pulp narrowing and calcification are other signs that the patient can present [4]. Enamel hypoplasia and delayed eruption can occur in children with chronic renal diseases [5, 28].

The oral condition of patients with renal failure has been described comprehensively in a recent meta-analysis by Ruospo et al. [29]. Authors clearly separated the prevalence of oral diseases in adults with ESRD, ESRD plus hemodialysis, and transplantation and explored any association between oral disease and mortality. They found that DMFT indices were similarly high in adults with chronic kidney disease (CKD) stages 1–5 (18.7 [C.I. 95% 10.5–27.0]) and those with CKD stage 5D (14.5 [C.I. 95% 12.7–16.3]), and the mean DMFT index increased with age but was not associated with gender or dialysis duration. Periodontitis affected 31.6% of adults with CKD stages 1–5 and 58% of patients in dialysis. The prevalence of periodontitis in stage 5D was unaffected by age, but increased with the proportion of women and duration of dialysis. The mean plaque index was 1.14 and 1.62 in two population with CKD stages 1–5 and 2.19 in kidney transplanted recipients. In stage 5D, the mean plaque index was 1.9 and increased with age but was not influenced by gender or time treated with dialysis. Regarding mucosal diseases, ulcerations affected 8.6% of patients in stage 5D and 1.3% of transplant recipients and candidiasis affected 22.2% of patients in stage 1–5, 19% of adults with CKD in stage 5D, and 13.3% of kidney-transplanted patients. The prevalence of oral candidiasis in stage 5 increased with age but not gender, time of dialysis, or geographical region. Xerostomia was reported by 48.4% of patients in stage 5D; the mean stimulated predialysis salivary flow rate was 0.86 ml/min for CKD stage 5D whereas the mean unstimulated salivary flow rate was 0.22 ml/min.

## 5. General Considerations for Dental Management

Patients with renal disease in conservative medical treatment or with PD do not generally require special measures regarding dental treatment, apart from avoiding nephrotoxic drugs (such as tetracyclines or aminoglycosides) and monitoring blood pressure during the procedures due to the frequent hypertension [5].

However, for hemodialysis patients, communication with the nephrologist is highly recommended in order to know the stage of the pathology, the medications prescribed, and comorbidities such as diabetes that negatively influence the homeostasis of these patients [5].

In diabetic dialysis patients, hypoglycemic agents and nutritional alterations can trigger hypoglycemia in the background of diminished gluconeogenesis, reduced insulin clearance by the kidney, and improved insulin sensitivity following initiation of renal replacement therapy. Detailed evaluation of antidiabetic regimen and nutritional patterns, patient education on self-monitoring of blood glucose, and/or referral to a diabetes specialist may reduce risk of subsequent hypoglycemia [30].

Other important features to take into consideration are drug intolerance and increased susceptibility to infection.

### 5.1. Risk of Bleeding

Dental treatment with risk of bleeding should be postponed to nondialysis day since the anticoagulant effect of heparin is absent, the bloodstream is free from toxic metabolites, and the patient is not debilitated by the treatment.

The administration of a heparin antagonist (protamine sulphate) can reduce the rate of bleeding in case of urgency. However, a persistent bleeding tendency remains due to anemia and alteration in platelet aggregation and adhesiveness. A hematologic study before planning any invasive treatment can give information about coagulation times, platelets count, hematocrit, and hemoglobin. In renal patients taking warfarin, International Normalized Ratio (INR) should be measured. Evidence-based medicine states that minor surgical procedure can be safely carried out without adjustment for INR<4 although for INR>2.5, a consultation with nephrologist is indicated [3, 31]. After the treatment, local hemostatic measures (compression, cold applications, tranexamic acid, cellulose sponges, and sutures) can be used in case of local hemorrhage and are generally sufficient to obtain hemostasis.

### 5.2. Medications

Local anesthetics can be safely used because they have a hepatic elimination. Paracetamol remains the best choice for pain management, and also codeine can be used without modification of the dosages. Other anti-inflammatory drugs such as ketoprofen, ibuprofen, or naproxen could cause hypertension and worsen the bleeding tendency. Aspirin is contraindicated because it increases platelet dysfunction, the risk of gastric hemorrhage, and contributes to the deterioration of renal function. In case of doubts, the nephrologist or the personal physician should be consulted.

Patients who have been treated with high doses of corticosteroids for a long time and or in stressful situations may require steroid supplementation prior to dental treatment to avoid an episode of adrenal crisis [5]. Moreover, it is suggested that dental sessions should take place in the morning, in a quiet environment and that abrupt and unexpected movements be avoided during therapy [3].

### 5.3. Antibiotic Prophylaxis and Therapy

Recent studies pointed out the lack of scientific evidence to prescribe antibiotic prophylaxis for preventing infective endocarditis (IE) in ESRD patients [15, 32]; ESRD or hemodialysis do not represent a criterion for IE prophylaxis. According to the American Heart Association guidelines, antibiotic administration remains indicated for patients suffering from concomitant cardiac comorbidities such as those with prosthetic cardiac valve, previous IE, unrepaired cyanotic congenital heart disease (CHD), completely repaired congenital heart defect with prosthetic material or device during the first six months after the procedure, repaired CHD with residual defects at the site or adjacent to the site of a prosthetic patch or prosthetic device, and cardiac transplantation recipients who develop cardiac valvulopathy [33]. Nevertheless, patients affected by ESRD have an increased susceptibility to IE especially if they do not have a good control of the disease [34]. Furthermore, patients in hemodialysis can develop infections of the vascular access (endarteritis) that can become itself the source of bacteraemia, and thus they may benefit from antibiotic prophylaxis, especially in the 6 months after the creation of the vascular access [35]. Due to these persisting controversies, the best practice remains the discussion with the patient's nephrologist to evaluate, case by case, the indication for an antibiotic prophylaxis.

In the presence of an acute or re-exacerbated dental infection (periapical periodontitis, periapical, or periodontal abscess), a complete cycle of antibiotic therapy should be administered using nonnephrotoxic antibiotics and taking into consideration the CC because as renal function is reduced, the plasma levels of some drugs may be high or prolonged [4]. The CC test assesses glomerular function comparing the amount of creatinine in the blood with that eliminated in the urine over a day. Theoretically, a 50% drop in CC represents a twofold increase in the half-life of a drug eliminated solely via renal excretion [9]. For this reason, according to the degree of renal elimination of the drug, the interval between doses should be increased.

Penicillin and its derivatives, clindamycin and cephalosporins are safer antibiotics for these patients [5]. Aminoglycosides, tetracyclines, and polypeptide antibiotics should be avoided because of their nephrotoxicity [4].

### 5.4. Psychological Aspect

Finally, it is extremely important to remember the psychological aspect in treating ESRD or hemodialyzed patients [4]. A poor quality of life and depression have been associated with hemodialysis, and a reduction of compliance should be expected in a higher percentage of patients compared with general population [36, 37].

However, it is debatable whether systemic alterations and general morbidity are casually associated with the worse dental and periodontal status or if the hemodialysis per se in combination with psychological factor may impact on quality of life [38].

Recently, Pakpour et al. investigated the oral health-related quality of life (OHRQoL) related to sociodemographic variables, clinical findings, cognitive variables, oral health behaviours, and general health-related quality of life (GHRQoL) in ESRD patients undergoing hemodialysis [39]. Patients were matched with a healthy control group. Results showed that patients on hemodialysis had poor oral health status, OHRQoL, and GHRQoL compared to healthy subjects. Sociodemographic variables, oral health knowledge, hygiene attitudes, and GHRQoL predicted OHRQoL.

Conversely, Schmalz et al. did not find neither clinical nor statistical significant differences of Oral Health Impact Profile (OHIP G14) between patients on hemodialysis and control group although ESRD patients exhibited worse oral health [38]. This result highlights that the patient's perception does not reflect the oral deficiencies and that education and motivation of these patients represent a focus in oral health maintenance.

## 6. Operative Protocols

An increased perception of the importance of oral health in ESRD patients and transplant candidates has been observed in the scientific community in the last twenty years. Over time, the dental protocols that have been proposed by various authors showed a growing attention to the psychological aspect of the patient and to the importance of maintaining good control of plaque and daily oral hygiene [5, 6, 9, 16, 31, 40]. Due to the greatly increased incidence and severity of periodontitis in the hemodialysis population, the dentist should keep in mind that the lack of oral hygiene may put the patient at higher risk of local or disseminated infection because of the persisting daily episodes of bacteraemia from the oral cavity. The spread of oral bacteria can be minimized by the elimination of oral foci and by reducing the grade of mucosal and gingival inflammation. A good control of oral hygiene and the absence of dental foci represent a fundamental step to receive a preemptive kidney transplant before the patient needing dialysis, if medically suitable, thanks to a living donor. Also, an efficient dental treatment with maintenance of good oral hygiene is essential in the posttransplant phase, especially by preventing the occurrence of severe infections and consequently the survival of the transplanted organ. Furthermore, adequate plaque removal and the treatment of gingivitis and periodontitis can avoid or minimize gingival hypertrophy due to assumption of immunosuppressive drugs such as cyclosporine [41].

Besides the knowledge of general health aspects discussed above, the dental practitioner has to know the correct practical approach and the operative sequences to follow when treating renal patients. From the first appointment, it is fundamental to impress upon the patient the importance of adequate oral health and explaining to them the possible complications arising from untreated oral foci, both in the pre- and posttransplantation phases, and the possible oral side effects of taking future antirejection therapy [16, 42, 43].

As already mentioned, an accurate medical history should be collected with particular reference to ESRD-related illnesses, medications and their dosage, blood parameters, timing, and type of dialysis performed. These aspects have to be directly discussed with the nephrologists when necessary [5, 44]. The dental exam consists of a noninvasive complete assessment of dental, periodontal, and mucosal tissues [9]. Special care should be taken when positioning the patient, avoiding compression of the arm with the vascular access for hemodialysis [5]. All possible foci (periodontal and endodontic lesions, residual roots, partially erupted and malpositioned third molars, and peri-implantitis) and oral pathologies (caries and mucosal lesions) have to be intercepted. Radiographs (orthopantomography and intraoral x-rays) complete the diagnostic process both in dentate and edentulous patients. Furthermore, a periodontal chart should be performed if periodontitis is suspected. The treatment plan for periodontal disease must include the assessment of the patient's oral hygiene. Therapy of gingivitis and periodontitis should consist primarily in accurate motivation and instruction for home oral hygiene, adapted and personalized to the necessities of the patient. Mechanical removal of supra- and subgingival calculus should be performed with ultrasound devices and curettes.

Carious lesions must be recognized and when necessary pulp vitality should be tested. In the presence of pulp necrosis and/or apical lesions, endodontic treatment, apicectomy, or extraction can be programmed. Generally, extractions are recommended when conservative, endodontic, and periodontal treatments do not guarantee the complete resolution of the pathology [3, 4, 20]. Extraction of partially erupted and malpositioned third molars is recommended to avoid pericoronal infection especially in the early posttransplant period. In cases of peri-implantitis, surgical removal of the implant should be performed. The surgery should be as atraumatic as possible to avoid maxillo-mandibular fractures due to renal osteodystrophy. In the presence of suspected mucosal lesions that do not resolve in 7–10 days, a biopsy must be performed. Before any procedure that could lead to bleeding (periodontal chart, calculus removal with ultrasound, subgingival scaling, extraction, and periodontal surgery) a 15 ml rinse of chlorhexidine 0.12% for 60 seconds is recommended to reduce the amount of oral bacteria that could reach the bloodstream. Adaptation of removable prostheses should be assessed to determine the necessity of adjustment or substitution, and the patient should be instructed regarding the cleaning and maintenance of the device [3].

Orthodontic appliances can be maintained if they do not interfere with oral hygiene. The removal of orthodontic brackets is indicated just before transplantation because the immunosuppressive therapy administered in the posttransplant period can induce gingival overgrowth that seems to be much more accentuated in the presence of fixed appliances [3].

After the first cycle of dental therapy, it is crucial that all patients are included in a strict follow-up program to maintain the results obtained and, above all, to maintain a high level of compliance. The frequency of follow-up depends on the needs and motivation of the patient (3–6 months for effective plaque control) [45]. Since the patients undergo hemodialysis with a frequency of about three times a week for approximately 2–4 hours per session (plus their other medical appointments), they could be psychologically affected and poorly compliant with respect to dental appointments. For this reason, the education on the importance of oral health is crucial to maintain the best motivation of the patient. At each appointment, a review of medical history is indicated and a complete noninvasive examination of the oral cavity should be repeated [15]. Radiographs must be performed if the presence of new foci is suspected.

It is important that this protocol is followed until the patient undergoes renal transplantation. In fact, the adherence to periodic follow-up reduces the risk of oral infection just before the transplant and avoids the possibility of losing the compatible organ because of an acute infection from a dental source [15].

**Figure 1:**
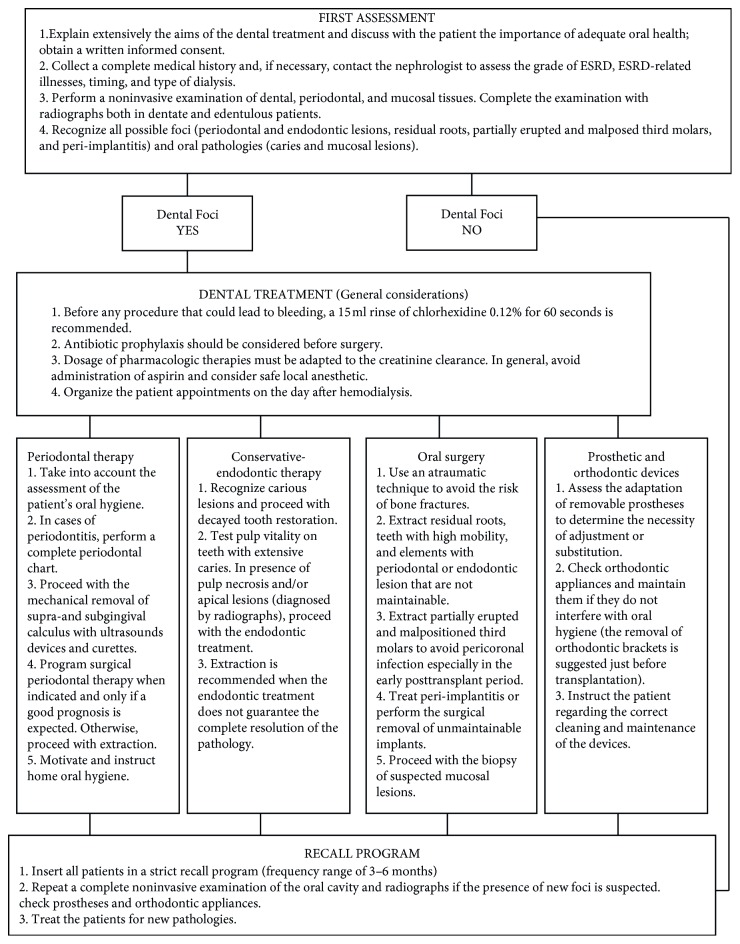
Flowchart for dental treatment of ESRD and hemodialyzed patients.

Operative protocols from first access to follow-up visit are summarized in Figure 1.

## 7. Conclusion

CKD has become a major public health problem worldwide, and terminal stage of CKD, ESRD, requires renal replacement therapy or kidney transplant [46, 47].

The number of patients needing a kidney transplant is increasing, and as this group of patients grows, its dental needs will also increase.

It is important to coordinate with the nephrologist the dental intervention considering the possible deterioration of general conditions of the patient during and after the dental treatment.

Patients have to be treated considering all issues correlated to the kidney dysfunction and inserted in a strict follow-up program until transplantation. Early detection of oral pathologies and strong preventive measures can minimize the need for extensive dental care [48]. The involvement of the patient is central to increase the motivation for oral health.
